# Control of Thousand-Grain Weight by *OsMADS56* in Rice

**DOI:** 10.3390/ijms23010125

**Published:** 2021-12-23

**Authors:** Zi-Wei Zuo, Zhen-Hua Zhang, De-Run Huang, Ye-Yang Fan, Si-Bin Yu, Jie-Yun Zhuang, Yu-Jun Zhu

**Affiliations:** 1 State Key Laboratory of Rice Biology, China National Rice Research Institute, Hangzhou 310006, China; zuoziwei@126.com (Z.-W.Z.); zhangzhenhua@caas.cn (Z.-H.Z.); huangderun@caas.cn (D.-R.H.); fanyeyang@caas.cn (Y.-Y.F.); 2 National Key Laboratory of Crop Genetic Improvement, College of Plant Science and Technology, Huazhong Agricultural University, Wuhan 430070, China; ysb@mail.hzau.edu.cn

**Keywords:** minor effect, grain weight, heading date, quantitative trait locus, rice, MADS-box

## Abstract

Grain weight and size are important traits determining grain yield and influencing grain quality in rice. In a previous study, a quantitative trait locus controlling thousand-grain weight (TGW) in rice, *qTGW10-20.8*, was mapped in a 70.7 kb region on chromosome 10. Validation of the candidate gene for *qTGW10-20.8*, *OsMADS56* encoding a MADS-box transcription factor, was performed in this study. In a near-isogenic line (NIL) population segregated only at the *OsMADS56* locus, NILs carrying the *OsMADS56* allele of IRBB52 were 1.9% and 2.9% lower in TGW than NILs carrying the *OsMADS56* allele of Teqing in 2018 and 2020, respectively. Using *OsMADS56* knock-out mutants and overexpression transgenic plants, *OsMADS56* was validated as the causal gene for *qTGW10-20.8*. Compared with the recipients, the TGW of the knock-out mutants was reduced by 6.0–15.0%. In these populations, decreased grain weight and size were associated with a reduction in the expression of *OsMADS56*. In transgenic populations of *OsMADS56* driven by a strong constitutive promoter, grain weight and size of the positive plants were significantly higher than those of the negative plants. Haplotype analysis showed that the Teqing-type allele of *OsMADS56* is the major type presented in cultivated rice and used in variety improvement. Cloning of *OsMADS56* provides a new gene resource to improve grain weight and size through molecular design breeding.

## 1. Introduction

Rice (*Oryza sativa* L.) is one of the most important food crops in China. More than 60% of the population relies on rice as the staple food. Thousand-grain weight (TGW) is one of the most important determinants of rice grain yield and is determined by grain length (GL), grain width (GW) and grain thickness. GL and GW are also important indicators for evaluating the appearance quality of rice. Cloning of quantitative trait loci (QTLs) for grain weight and size has an important role in the molecular breeding of high-yield superior-quality rice varieties.

Causal genes for 22 QTLs controlling grain weight and size have been cloned. Two of the genes regulated GL and GW without affecting TGW, including *GL7*/*GW7* [1,2] and *GS9* [3]. The other 20 genes all affected TGW. One gene, *GSA1*, had similar effects on GL and GW with the same allelic direction, resulting in a large effect on TGW [4]. Six genes, namely *GW2* [5], *TGW2* [6], *GS5* [7], *GSE5* [8], *GW6* [9] and *GW8* [10], mainly regulated TGW and GW. The other 13 genes, namely *qTGW1.2b* [11], *GS2*/*GL2* [12,13], *OsLG3* [14], *OsLG3b*/*qLGY3* [15,16], *GS3* [17], *SG3* [18], *GL3.1*/*qGL3* [19,20], *qTGW3* [21], *qGL5* [22], *TGW6* [23], *GW6a* [24], *GL6* [25] and *GLW7* [26], mainly regulated TGW and GL. These genes are distributed on all the rice chromosomes except chromosomes 4, 10, 11 and 12. Three of them, *GSE5*, *GLW7* and *OsLG3*, were cloned by using genome-wide association studies or Ho-LAMap [8,14,26]. The other 19 genes were all isolated by using map-based cloning.

TGW, GL and GW are quantitative traits controlled by a few major-effect QTLs and many minor-effect QTLs. Generally, major-effect QTLs are often detected repeatedly in multiple populations, while minor-effect QTLs are identified in fewer populations [27]. Among the 22 cloned QTLs for grain weight and size, only two, *qTGW1.2b* [11] and *SG3* [18], exhibited minor effects. There are two main reasons for difficulty in the cloning of minor-effect QTLs: (1) Minor-effect QTLs are sensitive to phenotypic measurement bias, making it difficult to narrow down the QTL region. (2) Minor effects between different alleles make it difficult to verify the causal genes using genetic complementation test. However, with the development of biotechnology, site-directed gene knock-out has become common [28]. Causal genes for minor-effect QTLs could be verified by using genome editing technology to create knock-out mutants for the candidate genes. Additionally, in the process of creating knock-out mutants of the target gene, it is also possible to obtain a novel allelic variation of the target gene [11]. Therefore, cloning minor-effect QTLs could provide genetic resources for broadening the genetic diversity of rice varieties.

MADS-box genes are a huge gene family of transcription factors that can be classified into two categories, type Ⅰ and type Ⅱ. The former is composed of ARG80/SRF-like genes in animals and fungi, also called M-type genes in plants; the latter includes MEF2-like genes in yeast and animals, also called MIKC-type genes in plants [29]. A total of 75 MADS-box genes have been identified in rice and are distributed on all 12 rice chromosomes. They play an important role in the structure and development of floral organs, flowering and seed development [30]. Among them, three genes, *OsMADS1* [15,16], *OsMADS51* [31] and *OsMADS87* [32], were reported to affect grain weight and size in rice.

In our previous study, one QTL, *qTGW10-20.8* conferring grain weight difference between *indica* rice restorer lines Teqing (TQ) and IRBB52, was mapped in a 70.7 kb region flanked by DNA markers Te20811 and Te20882 on the long arm of chromosome 10. One annotated gene, *Os10g0536100* encoding MIKC-type MADS-box protein OsMADS56, was the most likely candidate gene [33]. In this study, *OsMADS56* was confirmed as the causal gene underlying *qTGW10-20.8* by testing near-isogenic lines (NILs), *OsMADS56* knock-out mutants and transgenic plants of *OsMADS56* driven by a strong constitutive promoter. Knock-out of *OsMADS56* caused a decrease in grain size and a reduction in the expression of *OsMADS56*. Decreased grain size in the NILs was also associated with a reduction in the expression. Similarly, expressing *OsMADS56* with a strong constitutive promoter resulted in greater grain weight and size. Haplotype analysis showed that the TQ-type allele of *OsMADS56* is the major type presented in cultivated rice and used in variety improvement. Our study provides a new gene resource to improve grain weight and size through molecular design breeding and broaden the genetic diversity of rice varieties by using genome editing.

## 2. Results

### 2.1. Genetic Effect of OsMADS56 in NIL Populations Derived from Teqing/IRBB52

**Figure 1:**
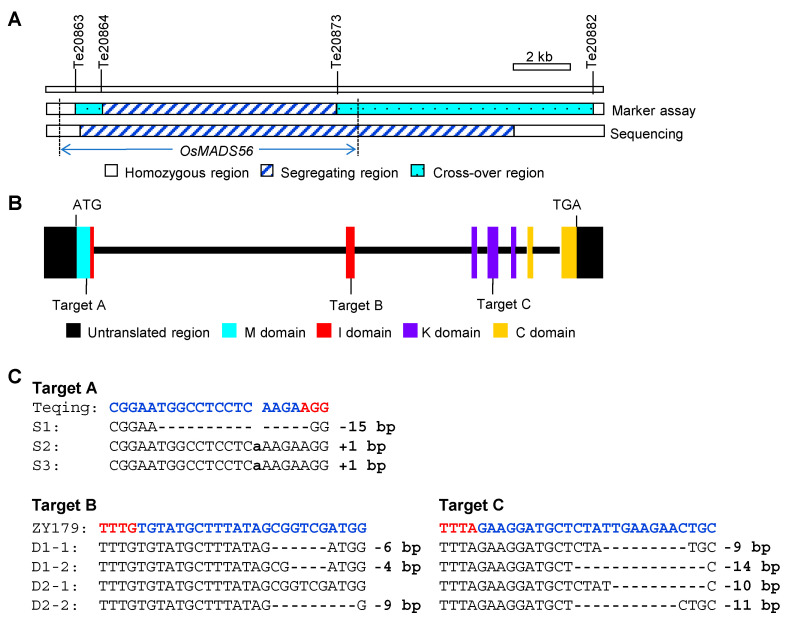
Variations of the mapping populations and *OsMADS56* knock-out mutants. (**A**) Same segregating regions existed in the two mapping populations, OS3 and TE2. They were determined by marker assay and sequencing. (**B**) Three target sites of *OsMADS56*. OsMADS56 contains four domains, i.e., MADS domain (M), intervening domain (I), keratin-like domain (K) and C-terminal domain (C). (**C**) Sequence variations of the mutations. S1, S2 and S3 were three homozygous mutants for target A in the Teqing background. D1-1, D1-2, D2-1 and D2-2 were four homozygous mutants for targets B and C in the ZY179 background. The target sequences and protospacer adjacent motif sites are shown in blue and red, respectively. Insertion is indicated by lowercase letter; deletion, by short dashes.

The first experiment to confirm whether *OsMADS56* is the target gene of *qTGW10-20.8* was conducted using two populations having the same segregating region that only involved one gene, *OsMADS56* (Figure 1A). They were derived from an F_13_ plant of the rice cross TQ/IRBB52 as illustrated in Figure S1. One population, OS3, was an NIL-F_2_ population consisting of 463 individuals. The other population, TE2, was an NIL population consisting of 31 NIL-TQ lines carrying the homozygous TQ allele of *OsMADS56* and 26 NIL-IR lines carrying the homozygous IRBB52 allele of *OsMADS56*. Whole genome re-sequencing validated that the NILs had an identical homozygous genotype in other genomic regions.

The OS3 population was grown in Lingshui (LS), Hainan Province, China. QTL analysis using QTL IciMapping 4.1 [34] detected significant genetic effects on all the four traits analyzed, heading date (HD), TGW, GL and GW (Table S1). Compared with the IRBB52 allele, the TQ allele reduced HD by 1.2 days and increased TGW, GL and GW by 0.35 g, 0.026 mm and 0.007 mm, respectively. The proportions of phenotypic variance explained (*R*^2^) for HD, TGW, GL and GW were 5.4%, 7.9%, 3.3% and 2.9%, respectively. Degree of dominance for the four traits ranged from −0.47 to 0.37, indicating that the genetic action mode of *OsMADS56* is basically additive.

The TE2 population was grown in Hangzhou (HZ), Zhejiang Province, China, in 2018 and 2020. Eight traits were analyzed, namely HD, TGW, GL, GW, number of panicles per plant (NP), number of spikelets per panicle (NSP), number of grains per panicle (NGP) and grain yield per plant (GY). Phenotypic distributions of these traits are shown in Figure S2. Differences between the two genotypic groups were observed in the distributions of HD, TGW, GL and GW in 2020. For NIL-TQ lines, HD values were concentrated in lower-value areas, whereas the values of TGW, GL and GW were gathered in higher-value areas. The same tendency was found for these traits in 2018, though the difference was less obvious. No clear differences between the two genotypic groups were observed for the remaining four traits, NP, NSP, NGP and GY.

Results of two-way analysis of variance (ANOVA) for the eight traits are presented in Table 1. In 2018, significant effects were detected for HD, TGW, GL, GW and NSP. The TQ allele reduced HD by 0.5 days and increased TGW, GL, GW and NSP by 0.22 g, 0.026 mm, 0.004 mm and 1.5, respectively. The *R*^2^ values were 17.2%, 30.7%, 20.2%, 6.1% and 6.7% for HD, TGW, GL, GW and NSP, respectively. In 2020, significant effects were detected for all traits except NP. The TQ allele reduced HD by 0.5 days and increased TGW, GL, GW, NSP, NGP and GY by 0.33 g, 0.039 mm, 0.013 mm, 2.6, 2.1 and 0.91 g, respectively. The *R*^2^ values were 23.6% for HD, 71.6% for TGW, 66.3% for GL, 35.8% for GW and 5.4–7.1% for other traits. These results indicate that *OsMADS56* may simultaneously affect multiple yield traits, but the major effect was on grain size and heading date.

### 2.2. OsMADS56 Knock-Out Mutants for Validating the Effects of OsMADS56

*OsMADS56* knock-out mutants were produced for validating the effects of *OsMADS56* on grain size and heading date. Three sites were targeted; they were located in the regions encoding three of the four OsMADS56 domains, namely MADS domain (M), intervening domain (I) and keratin-like domain (K) (Figure 1B). The oligonucleotide for target A was ligated into BGK03 vector (CRISPR/Cas9 system), and those for targets B and C were ligated into the same vector HS-lb4F (CRISPR/Cpf1 system). Two recipients were used. One was TQ, the parental line carrying the enhancing allele for grain size. The other was ZY179, a pure line selected from an F_10_ population of TQ/ IRBB52. This line carried TQ alleles at *OsMADS56*, *GS2*/*GL2*, *TGW2*, *OsLG3*, *OsLG3b*, *qTGW3*, *TGW6*, *GW6a*, *GL6*, *GLW7*, *GL7*/*GW7* and *GS9* and carried IRBB52 alleles at *GW2*, *GS3*, *SG3*, *GL3.1*/*qGL3*, *GSA1* and *GSE5*. By analyzing the sequences of *GS3* and *GSE5*, we found that *GS3*^IRBB52^ and *GSE5*^IRBB52^ alleles belonged to long-grain and narrow-grain types, respectively. While TQ and ZY179 both carried the *OsMADS56*^TQ^ allele for enhancing grain size, ZY179 is more slender than TQ, having a grain shape similar to NILs of the TE2 population (Figure S3).

Five independent T_0_ mutants were selected, including three homozygous mutants for target A in the TQ background and two biallelic mutants for targets B and C in the ZY179 background. In the two T_1_ populations of biallelic mutants, genotypes of targets B and C were completely cosegregated (data not shown), resulting in the identification of two homozygous mutation types in each population. Thus, a total of seven homozygous mutants were used for further analysis (Figure 1C). For target A, S1 had a 15-bp deletion, and S2 and S3 contained a 1-bp insertion at the same site. For targets B and C, D1-1 had a 6-bp deletion and a 9-bp deletion, D1-2 had a 4-bp deletion and a 14-bp deletion, D2-1 had no mutation and a 10-bp deletion, and D2-2 had a 9-bp deletion and an 11-bp deletion, respectively.

### 2.3. Genetic Effect of OsMADS56 in Knock-out Mutants

**Figure 2:**
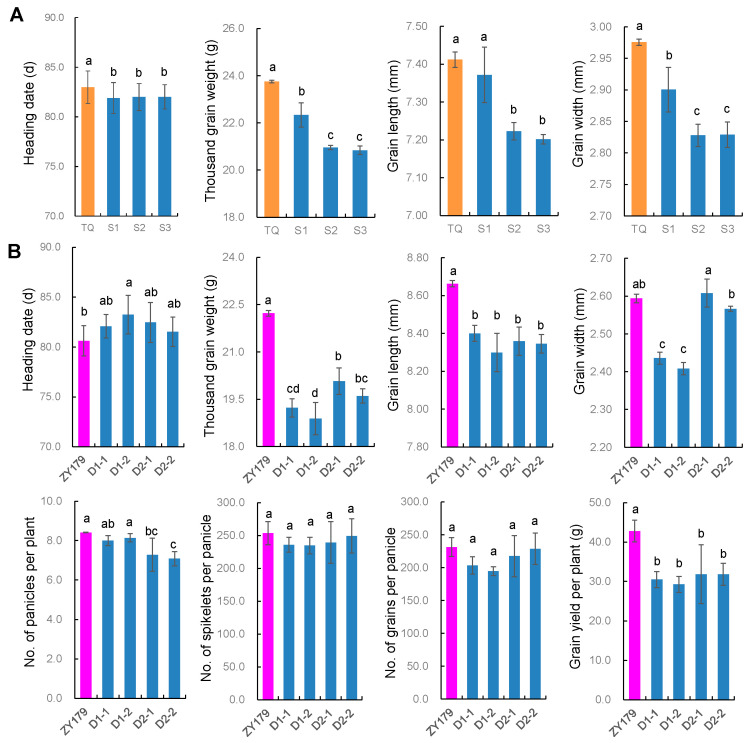
Phenotypic differences among each recipient and its mutants. (**A**) Heading date and grain size traits in Teqing and its mutants. (**B**) Heading date, grain size and yield traits in ZY179 and its mutants. Values are given as the mean ± SD (*n* = 3). Values with different letters are significantly different at *p* < 0.05 based on Duncan’s multiple range test.

Nine genotypes of the two knock-out populations were tested in HZ, in which each genotype included three lines. One population included TQ and its mutants S1, S2 and S3 at target A. The other comprised ZY179 and its mutants D1-1, D1-2, D2-1 and D2-2 at targets B and C. Eight traits were analyzed, among which HD, TGW, GL and GW were tested for all the rice lines and NP, NSP, NGP and GY were only examined for ZY179 and its mutants. Results on the phenotypic differences among each recipient and its mutants, tested by using Duncan’s multiple range test, are presented in Figure 2 and Table S2.

The three target-A mutants, S1, S2 and S3, all decreased from the recipient TQ in all the four traits tested, which were all significant except GL of S1 (Figure 2A). Decreases in HD were similar among the three mutants, ranging from 1.0 to 1.1 days. Decreases in the other three traits were also similar between the two mutants with the same 1-bp insertion, S2 and S3, estimated as 2.80 and 2.92 g for TGW, 0.189 and 0.210 mm for GL, and 0.148 and 0.147 mm for GW. On the other hand, decreases in TGW, GL and GW were much weaker in the mutant with a 15-bp deletion, S1, estimated as 1.42 g, 0.040 mm and 0.075 mm, respectively. In each mutant, percentage change was much higher in TGW than in other traits and higher in GW than in the remaining two traits. The values for TGW, GW, GL and HD were −6.0%, −2.5%, −0.5% and −1.3% in S1; −11.8%, −5.0%, −2.6% and −1.2% in S2; and -12.3%, −4.9%, −2.8% and −1.2% in S3, respectively (Table S2).

The four target-BC mutants, D1-1, D1-2, D2-1 and D2-2, all showed an increase in HD over the recipient ZY179, but only the change in D1-2 was significant (Figure 2B). On the other hand, the four mutants were decreased in grain size over ZY179. D1-1 and D1-2 showed significant decreases in all the three traits for grain size, and D2-1 and D2-2 were significantly decreased in TGW and GL but showed a nonsignificant change from ZY179 in GW. Changes in grain size traits were much more stable in TGW and GL than in GW. Among the four mutants, the values ranged from −2.15 to −3.34 g (−9.7% to −15.0%) for TGW, −0.263 mm to −0.364 mm (−3.0% to −4.2%) for GL, and 0.014 to −0.186 mm (0.5% to −7.2%) for GW (Table S2). In the other four yield traits, NP, NSP, NGP and GY, deceases from ZY179 were all observed in each mutant. The changes were all significant in GY and nonsignificant in NSP and NGP (Figure 2B). For NP, the changes were nonsignificant in D1-1 and D1-2 but significant in D2-1 and D2-2. These results indicate that *OsMADS56* may simultaneously affect multiple yield traits, but its influence on grain yield is mainly contributed by TGW.

Taking the results on HD and grain size in the two knock-out populations together, it is noted that the effect on TGW was the highest and most stable and the effect on HD was the lowest and least stable. The results also indicate that knock-out at target A had a larger effect on GW than on GL, whereas knock-out at targets B and C had a larger effect on GL than on GW.

### 2.4. Performance of Transgenic Plants Expressing OsMADS56 Driven by an Actin Promoter

Transgenic plants expressing the *OsMADS56* gene driven by an Actin promoter were also produced for validating the effects of *OsMADS56*. The full-length cDNA of *OsMADS56*^TQ^ was introduced into ZY180. ZY180 and the knock-out recipient ZY179 were identical except that ZY180 carried the low-expression *OsMADS56*^IRBB52^ allele and ZY179 had the high-expression *OsMADS56*^TQ^ allele.

**Figure 3:**
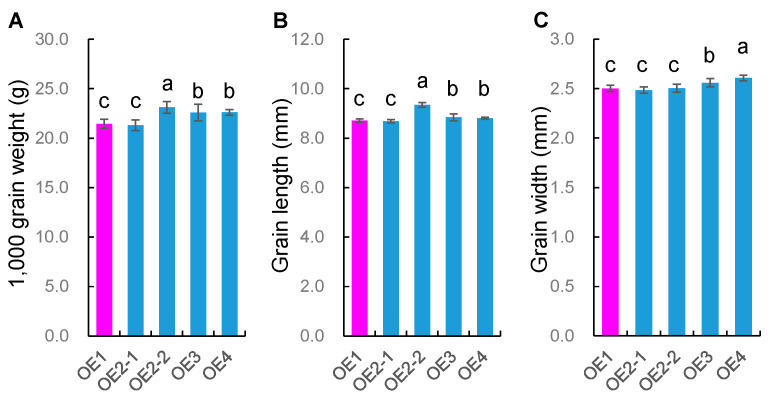
Phenotypic performance of *OsMADS56* driven by an Actin promoter. (**A**) Thousand-grain weight (g). (**B**) Grain length (mm). (**C**) Grain width (mm). Values are given as the mean ± SD (*n* = 24 for OE1, OE3 and OE4; *n* = 21 for OE2-1; *n* = 18 for OE2-2). Values with different letters are significantly different at *p* < 0.05 based on Duncan’s multiple range test.

Four T_2_ populations were tested in HZ, namely one transgenic negative control (OE1), one segregating population (OE2) and two positively homozygous lines (OE3 and OE4). Three traits, namely TGW, GL and GW, were measured. Results on the phenotypic differences among the four populations, tested by using Duncan’s multiple range test, are presented in Figure 3 and Table S3. Compared with the negative control, negative plants of the segregating population showed no significant difference, whereas positive plants of the segregating population and the two positively homozygous lines were significantly larger in grain weight and size. The increase ranged from 1.15 to 1.68 g (5.1% to 7.8%) for TGW and from 0.094 to 0.637 mm (1.1% to 7.3%) for GL. These results indicate that *OsMADS56* driven by a strong constitutive promoter could increase grain weight and size.

### 2.5. Expression of OsMADS56 in NILs and Knock-Out Mutants

**Figure 4:**
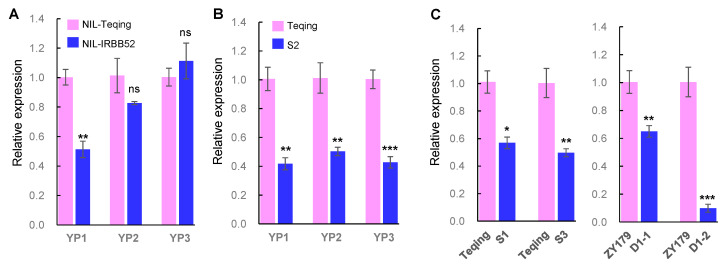
Relative expression of *OsMADS56* in young panicles of near-isogenic lines (NILs) and *OsMADS56* knock-out mutants determined by qRT-PCR. (**A**,**B**) Relative expression in three periods of the young panicles. YP1: panicles of 1–5 cm in length; YP2: panicles of 6–10 cm in length; YP3: panicles of 11–15 cm in length. NIL-Teqing and NIL-IRBB52 are near-isogenic lines carrying Teqing and IRBB52 alleles of *OsMADS56*, respectively. S2 is a mutant in the Teqing background. (**C**) Relative expression of four mutants in panicles at YP3. S1 and S3 are two mutants in the Teqing background. D1-1 and D1-2 are two mutants in the ZY179 background. Values are given as the mean ± SEM (*n* = 3). Significant difference was detected by using Student’s *t*-test. * *p* < 0.05, ** *p* < 0.01, *** *p* < 0.001, ns: not significant.

Quantitative real-time PCR (qRT-PCR) was used to analyze allelic differences of *OsMADS56* expression. The analysis was first performed using young panicles at three stages of panicle length (YP1, 1–5 cm; YP2, 6–10 cm; YP3, 11–15 cm) collected from NIL-TQ, NIL-IR, TQ and mutant S2. Relative expression of *OsMADS56* was not significantly different between NIL-TQ and NIL-IR at YP2 and YP3, but it was significantly higher in NIL-TQ than in NIL-IR at YP1 (Figure 4A). The significant difference is in the same direction as the grain size which was higher in NIL-TQ and lower in NIL-IR. For TQ and S2, the *OsMADS56* expression was significantly lower in S2 at all three stages (Figure 4B), which is also positively associated with the decreased grain size in S2. These results are also in accordance with the larger grain-size difference between TQ and S2 than between NIL-TQ and NIL-IR. Four more mutants, namely S1 and S2 from TQ and D1-1 and D1-2 from ZY179, were analyzed using samples at YP3. A significant decrease in the relative expression of *OsMADS56* was shown for all the mutants (Figure 4C). These results suggest that knock-out of *OsMADS56* will reduce its expression, causing a decrease in grain weight and size.

To further explore the regulatory mechanism of *OsMADS56*, RNA sequencing (RNA-seq) was used to analyze the expression levels of the cloned grain size genes and important heading date genes in three tissues, namely young panicles of 11–15 cm in length and penultimate leaves at 55 and 74 days after sowing (DAS).

Of the 22 cloned grain size genes in rice, none showed a significant difference between ZY179 and D1-2 or between NIL-TQ and NIL-IR in any of the three tissues analyzed. In sharp contrast, a significant difference in *OsMADS56* expression was found in all the comparisons involving the two pairs of genotypes, ZY179 vs. D1-2 and NIL-TQ vs. NIL-IR, in the three tissues (Figure S4). In all cases, the expression levels were higher in ZY179 than in D1-2 and higher in NIL-TQ than in NIL-IR. No other gene showed a significant difference between NIL-TQ and NIL-IR in the panicle tissue, but significant differences between ZY179 and D1-2 in this tissue were observed for four genes: *PhyB*, *HAPL1*, *OsLFL1* and *OsMADS15* (Figure S4A). Compared with ZY179, D1-2 showed a decrease in the expression of *PhyB* and *HAPL1* and an increase in the expression of *OsLFL1* and *OsMADS15*. Decreased expression levels of *PhyB* in D1-2 were also detected in the leaves at 55 DAS (Figure S4B) and 74 DAS (Figure S4C).

Among the comparisons on the six pivotal genes regulating heading date in rice, *Hd1*, *Ghd7*, *DTH8*, *Ehd1*, *RFT1* and *Hd3a*, significant differences in the expression were only found between ZY179 and D1-2 for *Ghd7* and *Hd3a* in leaves at 55 DAS (Figure S4B) and for *Ghd7* at 74 DAS (Figure S4C). In all cases, the expression was lower in ZY179 than in D1-2. Regarding the two florigen genes in rice, the expression of *RFT1* was much stronger than that of *Hd3a* at 55 DAS and 74 DAS in all the four rice lines, but no significant difference was found between ZY179 and D1-2 or between NIL-TQ and NIL-IR. To verify the results of RNA-Seq, expression levels of three genes (*OsMADS56*, *RFT1* and *Hd3a*) in the leaves at 55 DAS and 74 DAS were determined using qRT-PCR. Compared with ZY179, D1-2 showed a decrease in the expression of *OsMADS56* and an increase in the expression of *Hd3a* at 55 DAS and 74 DAS. The expression of *RFT1* was not significantly different between ZY179 and D1-2 (Figure S5). These results are consistent with those of RNA-seq.

### 2.6. Allelic Variation of OsMADS56 in Cultivated Rice and Its Association with HD and Grain Size

To study the allelic variation of *OsMADS56* in cultivated rice, data were downloaded from the Rice Variation Map v2.0 (http://ricevarmap.ncpgr.cn/v2, accessed on 16 July 2021). A 9-bp InDel and 11 single-nucleotide polymorphisms were detected in the coding region of *OsMADS56* in 3490 cultivated rice accessions, based on which 14 haplotypes were classified and ordered following the number of accessions contained (Table S4). By comparing our sequencing data of the two parental lines with these haplotypes, we found that the TQ and IRBB52 types of *OsMADS56* belong to Hap1 and Hap5, respectively. In the 3490 accessions, Hap1 had the highest proportion of 73.9% and Hap5 had a low proportion of 0.6%.

Then, we analyzed the allelic variation of *OsMADS56* in 299 rice cultivars selected from the National Mid-term Genebank for Rice at the China National Rice Research Institute (Table S5). They included 121 improved varieties and 86 landraces of *indica* rice in China and 92 accessions introduced from other countries. Five haplotypes, from Hap1 to Hap5, were found in these cultivars (Table S4). The largest group remained Hap1, or the TQ type, containing 242 cultivars (80.9%). The second largest group changed to Hap5, or the IRBB52 type, containing 33 cultivars (11.0%). The other three haplotypes had a total of 24 cultivars. The 121 improved varieties in China contained 115 (95.0%) Hap1 and 6 (5.0%) Hap5 cultivars. The 86 landraces in China contained 78 (90.7%) Hap1, 5 (5.8%) Hap2 and 3 (3.5%) Hap 5 cultivars. The cultivars from other countries contained all the five haplotypes, among which Hap1 and Hap5 were the largest and second largest groups, having 49 (53.3%) and 24 (26.1%) cultivars, respectively. The other three haplotypes had a total of 19 cultivars. Six of the exotic cultivars have been widely used in rice breeding and/or commercial rice production in China. They all belong to Hap1 or Hap5 (Table S5).

The 299 varieties were planted in LS and HZ. Four traits, HD, TGW, GL and GW, were measured. Phenotypic distributions of these four traits are shown in Figure S6. Compared with the largest group, Hap1, significant differences in heading date and grain size were detected for other haplotypes except for the smallest group Hap4 (Table S6). Between the two largest groups, Hap1 (TQ type) and Hap5 (IRBB52 type), significant differences were observed for HD in LS and GW in both locations. Compared with Hap5, Hap1 decreased in HD by 13.3 days and increased in GW by 0.141 and 0.140 mm.

## 3. Discussion

This study identified *OsMADS56* as the causal gene underlying a QTL for grain weight and size in rice. The effect was first confirmed using NIL populations segregated for *OsMADS56* only and then validated through gene target mutagenesis. *OsMADS56* encodes an MIKC-type MADS-box protein that contains four domains, i.e., M, I, K and C-terminal (C) domains. The M domain is highly conserved and required for DNA-binding, dimerization and nuclear localization activities [35]. The I domain is necessary for dimerization [36], and the K domain is responsible for protein interaction [37]. The C domain is the least conserved domain and is responsible for transcriptional activation and high-order complex formation [38,39,40]. In the populations we used to validate the effect of *OsMADS56*, coding sequence variations were related to all four domains. The 9-bp InDel between TQ and IRBB52 [33] was located in the region for the C domain. Targets A, B and C for *OsMADS56* knock-out were located in the regions for the M, I and K domains, respectively (Figure 1B). NIL-IR with the 9-bp deletion showed a significant decrease in grain weight and size compared with NIL-TQ (Table 1). Decreased grain weight and size were also observed in the seven knock-out mutants of *OsMADS56*^TQ^ (Figure 2), regardless of whether the mutation occurred at target A (mutants S1, S2 and S3), target C (D2-1) or both targets B and C (D1-1, D1-2 and D2-2). These results indicate that sequence change in the coding region involving any of the four domains might affect grain weight and size. We also found that the decrease in grain weight and size was associated with a reduction in the expression of *OsMADS56* estimated with qRT-PCR (Figure 4) and RNA-seq (Figure S4), which is similar with some of the genes cloned for grain weight and size, such as *GS5* [7], *GW8* [10], *GS2* [12], *GL2* [13], *GL3.1* [19] and *GLW7* [26].

Although the effect of *OsMADS56* on grain weight and size has not been reported before, the role of *OsMADS56* in regulating heading date in rice has been studied. Overexpression of *OsMADS56* resulted in delayed heading under long-day conditions through the *OsLFL1*-*Ehd1* pathway [41]. In our study, NILs and mutants were grown under natural long-day conditions in HZ for analyzing the relationship between heading date and the expression of *OsMADS56* and key genes for heading date in rice. RNA-seq analysis of leaf samples in the transition period from vegetative to reproductive phase showed that the expression of *OsMADS56* was significantly higher in NIL-TQ and ZY179 than in NIL-IR and D1-2, respectively (Figure S4B). It was also shown that the heading was more promoted in NIL-TQ and ZY179 than in NIL-IR and D1-2, respectively (Table 1, Figure 2B). The association of higher *OsMADS56* expression with earlier heading is different from that of Ryu et al. [41], although our results also indicate that *OsMADS56* plays a role in regulating rice heading. In addition, no significant difference was found in the expression of *Ehd1* and *Hd1*, implying that *OsMADS56* may be involved in other unknown pathways.

Genes for grain weight and size in rice have been subjected to high selection pressure in domestication and artificial breeding [23,42]. In our study, haplotype analysis of *OsMADS56* using data from the Rice Variation Map v2.0 (http://ricevarmap.ncpgr.cn/v2, accessed on 16 July 2021) identified 14 haplotypes in 3490 cultivated rice accessions, of which the *OsMADS56^T^^Q^* type (Hap1) occupied a major proportion of 73.9% and the *OsMADS56^IRBB52^* type (Hap5) had a low proportion of 0.6%. In a collection of 299 *indica* rice varieties, the proportion of Hap5 increased to 11.0%. Moreover, the 121 improved varieties used in the southern rice region of China contain 115 varieties of Hap1, 6 varieties of Hap5, and no variety of other haplotypes. Four of the six varieties belonging to Hap5 were originated from crosses involving Hap5 varieties bred by the International Rice Research Institute based on the pedigree information (https://www.ricedata.cn/variety, accessed on 20 July 2021). Yuxian 3 and Shuhui 527 were originated from IR24, which is the parental variety of IRBB52 sharing the same allele of *qTGW10-20.8*. Two other varieties, Xiangwanxian 1 and Ezao 6, were derived from IR22 and IR8, respectively. These results indicate that *OsMADS56* has been subjected to high selection pressure in modern rice breeding. Concentrated use of Hap1 and Hap5 also suggests that *OsMADS56* may play an important role in the adaptation of rice varieties.

Genetic variation is the essential resource for crop improvement, but it has been greatly reduced in the process of domestication and artificial selection [43,44]. Low genetic diversity has become the main bottleneck for rice improvement. In previous studies, we found that novel allelic variation with large phenotypic variation could be obtained by directional editing of minor-effect QTLs with genome editing technology [11]. NIL-IR was reduced by 1.9% and 2.9% for TGW in 2018 and 2020 compared with NIL-TQ, respectively. The knock-out lines were reduced by 6.0–12.3% and 9.7–15.0% for TGW compared with TQ and ZY179, respectively. These results provide another piece of evidence for the potential of utilizing minor-effect QTLs in rice breeding through genome editing.

## 4. Materials and Methods

### 4.1. Plant Materials

Three sets of rice materials were used in this study. The first one contained an NIL-F_2_ and an NIL population that varied in the *OsMADS56* gene region only, the second contained *OsMADS56* knock-out lines in two genetic backgrounds and the last contained transgenic plants of *OsMADS56* driven by an *Actin* promoter in one genetic background.

The NIL-F_2_ and NIL populations were derived from an F_13_ plant of the rice cross TQ/IRBB52. This plant had a single heterozygous region, Te20864–Te20873 on the long arm of chromosome 10, which involved only one gene, *OsMADS56*. One NIL-F_2_ population only segregated in the *OSMADS56* gene region was produced, namely OS3. Nonrecombinant homozygotes were selected from this population and selfed to produce an NIL population, namely TE2 (Figure S1).

*OsMADS56* knock-out mutants in the backgrounds of TQ and ZY179 were generated by using CRISPR/Cas9 and CRISPR/Cpf1 systems, respectively. Five independent T_0_ mutants were selected, including three homozygous mutants for target A in the TQ background and two biallelic mutants for targets B and C in the ZY179 background. Transgenic plants of *OsMADS56* driven by an *Actin* promoter were produced in the ZY180 background. Four independent T_0_ transgenic plants were obtained, of which one was negative and the other three were positive. Each T_0_ plant was selfed to produce a T_1_ population. Individual T_1_ plants were genotyped and harvested. The T_2_ populations were used to verify that *OsMADS56* is the gene for *qTGW10-20.8*.

### 4.2. DNA Marker Analysis for QTL Mapping

DNA was extracted from 2 cm long leaves using a mini-preparation method [45]. PCR amplification followed the method of Chen et al. [46]. The products were separated on 6% nondenaturing polyacrylamide gels and visualized using silver staining. The markers (Table S7) were developed according to whole genome resequencing of TQ and IRBB52.

### 4.3. Construction of the Knock-Out and Overexpression Vectors

For knock-out experiment, three target sites were designed applying the web-based tool CRISPR-GE (https://skl.scau.edu.cn, accessed on 1 July 2017). Target A was located at +73 to +91 in the 1st exon, Target B was located at +197 to +220 in the 2nd exon and Target C was located at +349 to +372 in the 4th exon. Oligonucleotide Cri-56A (Table S7) for target A was ligated into the CRISPR/Cas9 expression vector BGK03 following the manufacturer’s instructions (Biogle Co., Ltd., Hangzhou, China). The original BGK03 vector comprised a rice U6 promoter for activating the target site sequence, a Cas9 gene driven by the maize ubiquitin promoter and a hygromycin marker gene driven by Cauliflower mosaic virus 35S promoter. Oligonucleotide Cri-56B and Cri-57C (Table S7) for targets B and C were ligated together into the CRISPR/Cpf1 expression vector HS-lb4F, which was constructed by BioRun Co., Ltd., Wuhan, China. The original HS-lb4F vector contains two minimal CaMV 35S promoters for activating the target site sequences, an LbCpf1 gene driven by CaMV 35S promoter and a hygromycin marker gene driven by CaMV 35S promoter (enhanced). The expression vectors were introduced into TQ and ZY179 using *Agrobacterium tumefaciens*-mediated transformation.

For the overexpression experiment, the full-length cDNA of *OsMADS56*^TQ^ was amplified from ZY179 using the marker OE-56 (Table S7) and cloned into the pCAMBIA2300 vector. The original pCAMBIA2300 vector comprised a rice *Actin* promoter for activating *OsMADS56* and a neomycin marker gene driven by CaMV 35S promoter. The overexpression vector was introduced into ZY180 using *Agrobacterium tumefaciens*-mediated transformation.

### 4.4. Detection of Transgenic Plants

Genomic DNA of the T_0_ plants was extracted from young leaves using the DNeasy Plant Mini Kit (Qiagen, Hilden, Germany). For the knock-out experiment, positive transgenic plants were identified by using the hygromycin gene marker Hyg. To identify mutations in the target region, the genomic fragment was amplified using the sequencing markers KO-56A, KO-56B and KO-56C. Mutation types of the five independent T_0_ mutants were confirmed by sequence analysis using the web-based tool DSDecodeM (http://skl.scau.edu.cn/dsdecode, accessed on 15 January 2018). For the overexpression experiment, genotypes of the transgenic plants were identified using the neomycin gene marker Neo and the *OsMADS56* gene marker Exon-56. Primers for these markers are listed in Table S7.

### 4.5. Field Experiments and Phenotyping

All the rice materials were grown at a spacing of 16.7 cm between plants and 26.7 cm between rows. Field management followed local agricultural practice. The 463 plants of the NIL-F_2_ population OS3 were grown in LS and measured on a single-plant basis. The 57 lines of the NIL population TE2 and the 27 lines of the *OsMADS56* knock-out populations were grown in HZ, following a randomized complete block design with two replications. For each replication, eight plants per line were planted in one row. HD was recorded for each plant and averaged for each replication. The middle five plants in each row were harvested at maturity and measured for NP, NSP, NGP and GY. Fully filled grains were selected and measured for TGW, GL and GW following the procedure reported by Zhang et al. [47]. The four populations of the *OsMADS56* overexpression experiment were grown in HZ and measured based on a single-plant basis. Each of the three homozygous populations contained 24 individual plants. The segregating population OE2 contained 39 plants, of which 18 were transgenic positive and 21 were transgenic negative.

### 4.6. Data Analysis

For the NIL-F_2_ population, QTL analysis was performed with the inclusive composite interval mapping (ICIM) of QTL IciMapping 4.1 [34]. The logarithm of the odds thresholds was calculated based on 1,000 permutations (*p* < 0.05) and used to declare a putative QTL. For the NIL population, two-way ANOVA was used to test phenotypic differences with the SAS procedure GLM (SAS Institute, Cary, NC, USA) following the method of Dai et al. [48]. When significant differences (*p* < 0.05) were found, the additive effects (*A*) and the proportions of phenotypic variance explained (*R*^2^) were calculated with the same model. For the knock-out mutants and recipients, Duncan’s multiple range test was employed to determine the phenotypic differences (*p* < 0.05).

### 4.7. RNA Extraction and Quantitative Real-Time PCR Analysis

Young panicles in three periods (1–5 cm, 6–10 cm and 11–15 cm) were collected from NIL-TQ, NIL-IR, TQ and S2. Young panicles in one period (11–15 cm) were collected from TQ, ZY179, S1, S2, D1-1 and D1-2. Total RNA was extracted using RNeasy Plus Mini Kit (Qiagen, Hilden, Germany). The first-strand complementary DNA was synthesized using ReverTra AceR Kit (Toyobo, Osaka, Japan). qRT-PCR was performed on Applied Biosystems 7500 using SYBR qPCR Mix Kit (Toyobo, Osaka, Japan). *Actin* was used as the endogenous control. Relative gene expression was analyzed according to the 2^−ΔΔCt^ method [49]. Three biological replicates were performed for each line, and three technical replicates were performed for each sample. The qRT-PCR primers are listed in Table S7.

### 4.8. RNA-Seq Analysis

Three tissues, 11–15 cm long young panicles, penultimate leaves at 55 DAS and penultimate leaves at 74 DAS, were collected from NIL-TQ, NIL-IR, ZY179 and D1-2 with four biological replicates. The 55 DAS is equivalent to the transition period from vegetative to reproductive phase, and 74 DAS is equivalent to the stage of young panicles in the length of 11–15 cm. RNA extraction, library preparation for transcriptome sequencing, data analysis and quality control were conducted by LC-Bio Technologies (http://www.lc-bio.com, accessed on 15 November 2020) according to their protocol. The total RNA was extracted using TRIzol reagent (Invitrogen, Carlsbad, CA, USA) following the manufacturer’s procedure. Quantity and purity of the total RNA were analyzed by using Bioanalyzer 2100 and RNA 6000 Nano LabChip Kit (Agilent, CA, USA). RNA-sequencing analysis was performed on an Illumina Hiseq2000/2500 (LC Sciences, CA, USA). The sequence results were presented as the FPKM (fragment per kilobase of exons per million reads). Differential expression analyses between NIL-TQ and NIL-IR and between ZY179 and D1-2 were performed using the Student’s *t*-test (*p* < 0.05).

### 4.9. Haplotype Analysis of OsMADS56

A total of 299 rice germplasms were used for haplotype analysis. Genomic DNA of the accessions was extracted using the method of Zheng et al. [45]. Three pairs of primers, namely Seq56-1, Seq56-2 and Seq56-3 (Table S7), were designed according to the sequence of *OsMADS56*^TQ^ that has been determined previously [33]. Three DNA fragments were amplified using these primers, which covered all the seven exons of *OsMADS56* completely and a small proportion of the introns. Sequences of the PCR products were determined using Sanger sequencing and the full-length cDNA sequence was assembled. Sequences were aligned by Clustal W program (University College Dublin, Dublin, Ireland) and analyzed with MEGA 6.0 software (Mega Ltd., Auckland, New Zealand). TQ and IR24 were included in the 299 germplasms. Additionally, the *OsMADS56* haplotype of IRBB52 was confirmed using Sanger sequencing.

## 5. Conclusions

Cloning of *OsMADS56* provides a new gene resource for improving grain weight and size through molecular design breeding. Novel alleles for *OsMADS56* with large phenotypic variation were generated using genome editing techniques, offering an effective approach to broaden the genetic diversity of rice varieties by using genome editing of minor-effect QTLs.

## Figures and Tables

**Table 1:** Effects of *OsMADS56* detected in near-isogenic lines.

Year	Trait ^a^	Phenotype (Mean ± SD) ^b^		*A* ^c^	*R*^2^(%) ^d^
		NIL-TQ	NIL-IR		
2018	HD	82.6 ± 0.8	83.7 ± 1.2	−0.5 ****	17.2
	TGW	23.84 ± 0.28	23.39 ± 0.20	0.22 ****	30.7
	GL	8.890 ± 0.036	8.838 ± 0.038	0.026 ****	20.2
	GW	2.563 ± 0.013	2.555 ± 0.014	0.004 **	6.1
	NP	9.1 ± 0.7	9.2 ± 0.7	n.s.	
	NSP	244.2 ± 11.5	241.1 ± 14.9	1.5 **	6.7
	NGP	194.9 ± 10.2	203.4 ± 14.9	n.s.	
	GY	38.55 ± 3.15	39.63 ± 4.01	n.s.	
2020	HD	82.3 ± 0.6	83.3 ± 0.8	−0.5 ****	23.6
	TGW	23.27 ± 0.18	22.60 ± 0.21	0.33 ****	71.6
	GL	8.746 ± 0.021	8.668 ± 0.029	0.039 ****	66.3
	GW	2.624 ± 0.016	2.597 ± 0.019	0.013 ****	35.8
	NP	8.9 ± 1.0	8.9 ± 1.0	n.s.	
	NSP	202.2 ± 11.0	197.0 ± 13.1	2.6 ****	6.6
	NGP	183.6 ± 9.8	179.4 ± 12.7	2.1 ****	5.4
	GY	36.64 ± 3.50	34.81 ± 3.77	0.91 ****	7.1

^a^ HD, heading date (days); TGW, 1000-grain weight (g); GL, grain length (mm); GW, grain width (mm); NP, number of panicles per plant; NSP, number of spikelets per panicle; NGP, number of grains per panicle; GY, grain yield per plant (g). ^b^ NIL-TQ and NIL-IR are near-isogenic lines carrying the Teqing and IRBB52 alleles of *OsMADS56*, respectively. ^c^ Additive effect of replacing an IRBB52 allele with a Teqing allele. n.s., nonsignificant; ** *p* < 0.01; **** *p* < 0.0001. ^d^ Proportion of phenotypic variance explained by the QTL effect.

## Data Availability

The datasets supporting the conclusions of this article are included within the article (and its additional files).

## Supplementary Materials

The following are available online at https://www.mdpi.com/article/10.3390/ijms23010125/s1.

## Abbreviations

ANOVA	Analysis of Variance
HD	Heading Date
GL	Grain Length
GW	Grain Width
GY	Grain Yield per Plant
NGP	Number of Grains per Panicle
NSP	Number of Spikelets per Panicle
NIL	Near-Isogenic Line
NP	Number of Panicles per Plant
QTL	Quantitative Trait Locus
R^2^	Proportion of Phenotypic Variance Explained
TGW	1000-Grain Weight
TQ	Teqing
