# Supplementary material for: Control of Thousand-Grain Weight by OsMADS56 in Rice
Source: Int J Mol Sci. 2021 Dec 23;23(1):125. doi: 10.3390/ijms23010125 (PMC8745348; doi:10.3390/ijms23010125)
Supplement: Supplementary file 1 [file ijms-23-00125-s001.zip › Supplementary Figures.pdf]

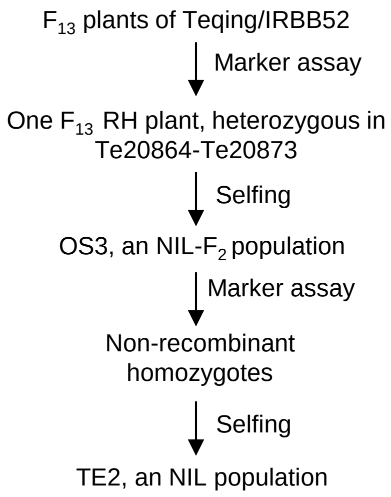

Figure S1. Development of the rice mapping populations used in this study. RH: residual heterozygote; NIL: near isogenic line.

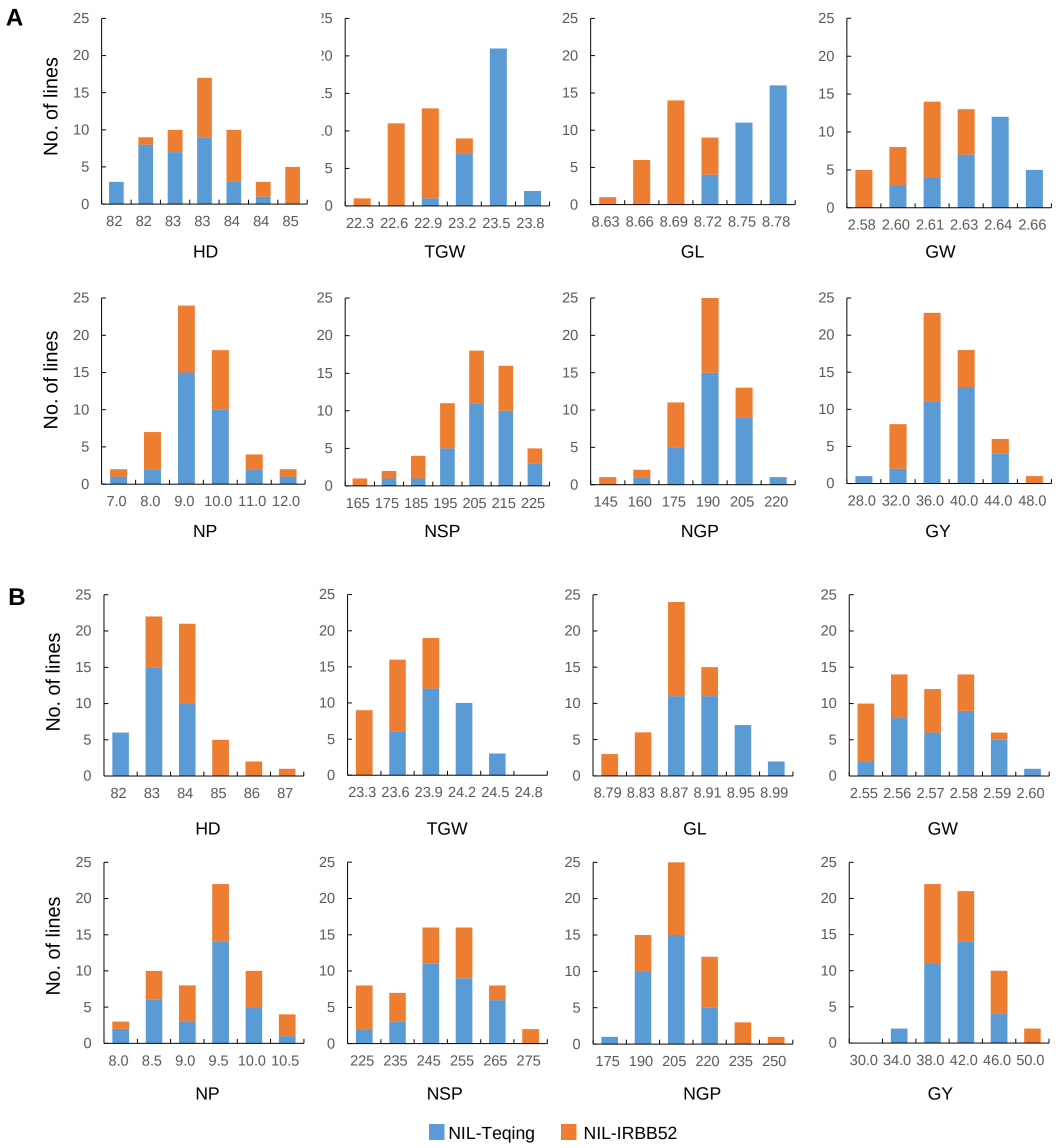

Figure S2. Phenotypic distributions of eight traits in the TE2 population. (**A**) In 2020. (**B**) 2018. HD, heading date (d); TGW, 1,000 grain weight (g); GL, grain length (mm); GW, grain width (mm); NP, number of panicles per plant; NSP, number of spikelets per panicle; NGP, number of grains per panicle; GY, grain yield per plant (g); NIL, near isogenic line.

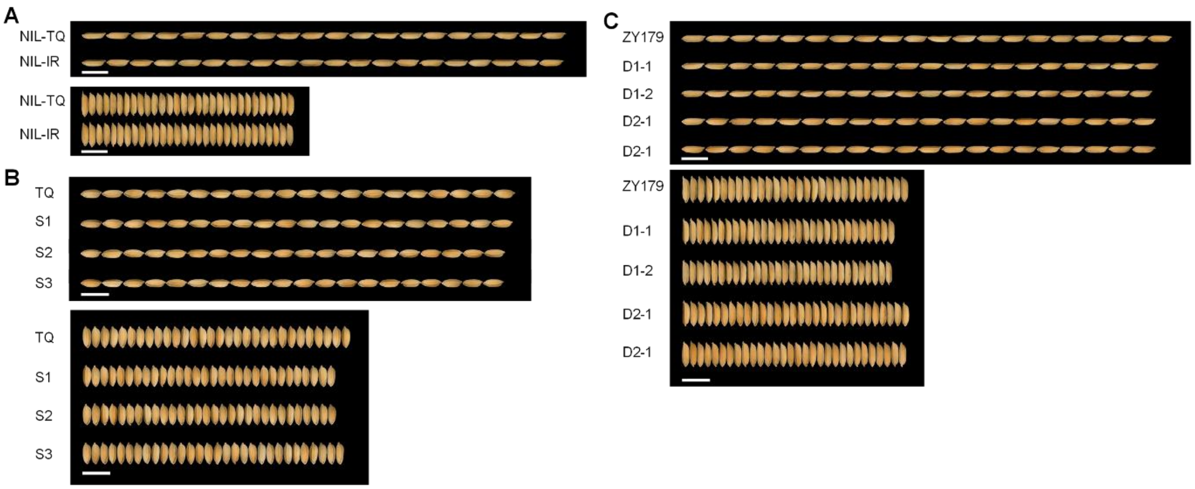

Figure S3. The effects of *OsMADS56* on grain length and grain width. (**A**) Comparisons of grain length and grain width in near isogenic lines. NIL-TQ and NIL-IR are near-isogenic lines carrying Teqing and IRBB52 alleles of *OsMADS56*, respectively. (**B**) Comparisons of grain length and grain width between Teqing and its mutants. (**C**) Comparisons of grain length and grain width between ZY179 and its mutants. Scale bars = 10 mm.

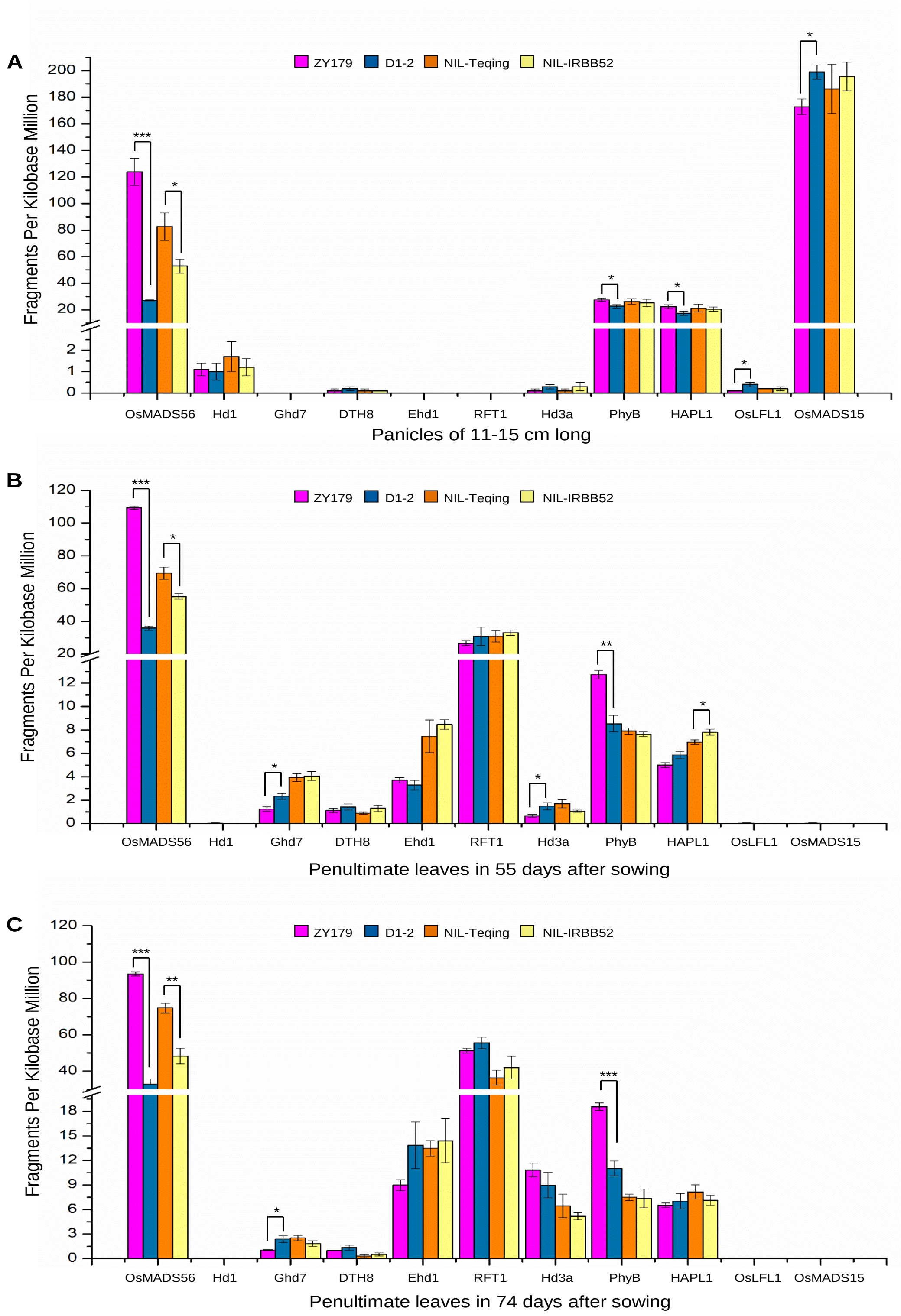

Figure S4. RNA sequencing analysis for two pairs of genotypes using three tissues. (**A**) Young panicles of 11-15 cm long. (**B**) Penultimate leaves in 55 days after sowing. (**C**) Penultimate leaves in 74 days after sowing. Values are given as the mean ± SEM ($n = 4$). Significant difference was detected by using Student's t-test. * $p < 0.05$, ** $p < 0.01$, *** $p < 0.001$.

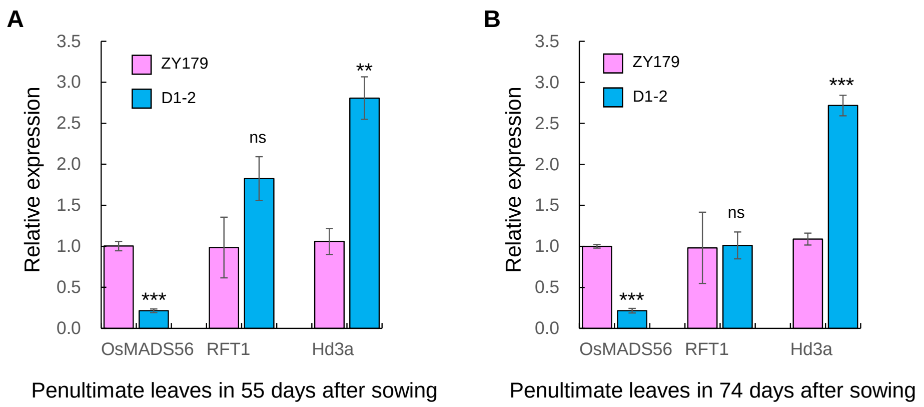

Figure S5. Relative expression of *OsMADS56*, *RFT1* and *Hd3a* in leaves of *OsMADS56* knock-out mutants determined by qRT-PCR. (**A**) Penultimate leaves in 55 days after sowing. (**B**) Penultimate leaves in 74 days after sowing. Values are given as mean ± SEM (n = 3). Significant difference was detected by using Student's *t*-test. * $p < 0.05$, ** $p < 0.01$, *** $p < 0.001$, ns: not significant.

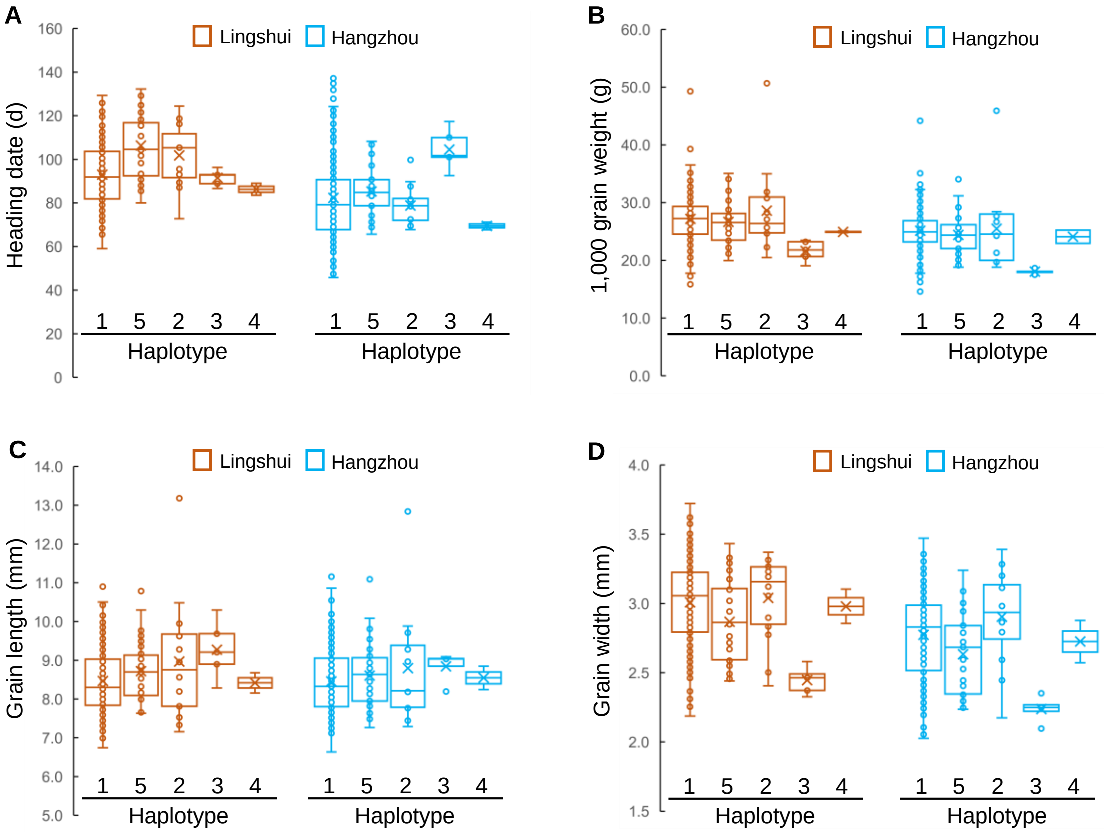

Figure S6. Phenotypic distribution of each haplotype in Lingshui and Hangzhou. (**A**) Heading date (d). (**B**) 1,000 grain weight (g). (**C**) Grain length (mm). (**D**) Grain width (mm).
