# Supplementary material for: Control of Thousand-Grain Weight by OsMADS56 in Rice
Source: Int J Mol Sci. 2021 Dec 23;23(1):125. doi: 10.3390/ijms23010125 (PMC8745348; doi:10.3390/ijms23010125)
Supplement: Supplementary file 1 [file ijms-23-00125-s001.zip › Supplementary Tables.pdf]

Table S1. Effects of *OsMADS56* detected in the NIL-F_2 population.

Trait[a]	Phenotype (mean ± SD)			*LOD*	A[b]	D[c]	$D/[A]$[d]	R^2(%)[e]
	Teqing	IRBB52	Heterozygote					
HD	107.0 ± 3.5	109.3 ± 3.3	108.5 ± 3.4	5.5	-1.2	0.4	0.32	5.4
TGW	26.69 ± 0.69	25.99 ± 0.88	26.34 ± 0.83	8.2	0.35	-0.002	-0.01	7.9
GL	9.237 ± 0.102	9.184 ± 0.091	9.220 ± 0.099	3.5	0.026	0.010	0.37	3.3
GW	2.771 ± 0.030	2.756 ± 0.029	2.760 ± 0.031	2.9	0.007	-0.003	-0.47	2.9

[a] HD, heading date (d); TGW, 1,000-grain weight (g); GL, grain length (mm); GW, grain width (mm). [b] Additive effect of replacing an IRBB52 allele with a Teqing allele. [c] Dominance effect. [d] Degree of dominance. [e] Proportion of phenotypic variance explained by the genetic effect.

Table S2. Heading date and grain size traits in recipients and knock-out lines of *OsMADS56*.

Name	Heading date (d)			1,000-grain weight (g)			Grain length (mm)			Grain width (mm)		
	Mean ± SD[a]	±RP[b]	±%[c]	Mean ± SD	±RP	±%	Mean ± SD	±RP	±%	Mean ± SD	±RP	±%
Teqing	83.0 ± 0.2 a			23.75 ± 0.05 a			7.412 ± 0.020 a			2.976 ± 0.005 a		
S1	81.9 ± 0.4 b	-1.1	-1.3	22.34 ± 0.51 b	-1.42	-6.0	7.372 ± 0.073 a	-0.040	-0.5	2.900 ± 0.035 b	-0.075	-2.5
S2	82.0 ± 0.2 b	-1.0	-1.2	20.96 ± 0.09 c	-2.80	-11.8	7.223 ± 0.023 b	-0.189	-2.6	2.828 ± 0.018 c	-0.148	-5.0
S3	82.0 ± 0.4 b	-1.0	-1.2	20.84 ± 0.18 c	-2.92	-12.3	7.202 ± 0.012 b	-0.210	-2.8	2.829 ± 0.020 c	-0.147	-4.9
ZY179	80.6 ± 0.5 b			22.22 ± 0.09 a			8.663 ± 0.016 a			2.594 ± 0.011 ab		
D1-1	82.1 ± 0.1 ab	1.5	1.8	19.23 ± 0.29 cd	-3.00	-13.5	8.400 ± 0.042 b	-0.263	-3.0	2.436 ± 0.016 c	-0.158	-6.1
D1-2	83.3 ± 0.9 a	2.6	3.3	18.89 ± 0.51 d	-3.34	-15.0	8.299 ± 0.102 b	-0.364	-4.2	2.408 ± 0.016 c	-0.186	-7.2
D2-1	82.3 ± 2.0 ab	1.6	2.0	20.07 ± 0.42 b	-2.15	-9.7	8.359 ± 0.075 b	-0.304	-3.5	2.608 ± 0.037 a	0.014	0.5
D2-2	81.6 ± 0.6 ab	0.9	1.2	19.60 ± 0.23 bc	-2.62	-11.8	8.345 ± 0.049 b	-0.318	-3.7	2.566 ± 0.007 b	-0.027	-1.1

[a] Numbers with different letters are significantly different at $p < 0.05$ based on Duncan's multiple range test. [b] Increase or decrease over Teqing or ZY179. [c] Increase or decrease over Teqing or ZY179 (%).

Table S3. Grain size traits in the overexpression populations of *OsMADS56*.

Name	Transgenic plants	*n*	1,000 grain weight (g)			Grain length (mm)			Grain width (mm)		
			Mean ± SD[a]	±ck[b]	±%[c]	Mean ± SD	±ck	±%	Mean ± SD	±ck	±%
OE1	Negative	24	21.44±0.46 c			8.713±0.072 c			2.502±0.031 c		
OE2-1	Negative	21	21.31±0.55 c	-0.13	-0.6	8.682±0.068 c	-0.031	-0.4	2.486±0.033 c	-0.016	0.01
OE2-2	Positive	18	23.12±0.60 a	1.68	7.8	9.350±0.093 a	0.637	7.3	2.504±0.040 c	0.002	0.0001
OE3	Positive	24	22.59±0.84 b	1.15	5.4	8.841±0.150 b	0.128	1.5	2.560±0.041 b	0.058	2.3
OE4	Positive	24	22.61±0.29 b	1.17	5.1	8.807±0.048 b	0.094	1.1	2.609±0.028 a	0.107	4.3

[a] Numbers with different letters are significantly different at $p < 0.05$ based on Duncan's multiple range test. [b] Increase or decrease over the transgenic negative control OE1. [c] Increase or decrease over the transgenic negative control OE1 (%).

Table S4. Haplotypes of *OsMADS56* in rice germplasms.

Haplotype	SNP1 162	SNP2 234	SNP3 288	SNP4 333	SNP5 356	SNP6 411	SNP7 443	SNP8 603	SNP9 608	SNP10 609	Indel 616-624	SNP11 669	NoV1[a]	NoV2[b]				Place of the two parents
														ImV	Lr	Ex	Total	
Hap1	C	C	T	A	G	C	G	C	C	A	GCGCCGCCGA	T	2579	115	78	49	242	Teqing
Hap2	C	C	C	A	T	G	A	C	C	A	GCGCCGCCGA	T	710	0	5	11	16	
Hap3	T	C	C	A	G	C	G	A	C	A	GCGCCGCCGA	T	84	0	0	5	5	
Hap4	C	C	C	A	G	C	A	C	C	A	GCGCCGCCGA	T	70	0	0	3	3	
Hap5	C	C	C	A	G	C	G	C	C	A	G	T	20	6	3	24	33	IRBB52
Hap6	C	C	C	A	G	C	G	C	C	A	GCGCCGCCGA	T	11	0	0	0	0	
Hap7	C	A	C	G	G	C	A	C	C	A	GCGCCGCCGA	T	5	0	0	0	0	
Hap8	C	C	C	A	G	C	A	C	G	C	GCGCCGCCGA	G	3	0	0	0	0	
Hap9	C	C	C	A	T	G	A	C	G	C	GCGCCGCCGA	G	2	0	0	0	0	
Hap10	C	C	C	A	G	C	A	C	C	A	GCGCCGCCGA	G	2	0	0	0	0	
Hap11	C	C	C	A	T	G	G	C	C	A	GCGCCGCCGA	T	1	0	0	0	0	
Hap12	T	C	C	A	T	G	A	C	C	A	GCGCCGCCGA	T	1	0	0	0	0	
Hap13	C	C	T	A	G	C	A	C	C	A	GCGCCGCCGA	T	1	0	0	0	0	
Hap14	C	C	T	A	T	G	A	C	C	A	GCGCCGCCGA	T	1	0	0	0	0	

[a] Number of varieties in 3490 germplasms. [b] Number of varieties in 299 germplasms. ImV, Improved variety in China; Lr, Landrace in China; Ex, Exotic germplasm.

Table S5. Rice germplasms used in this study.

Name	Origin	Type[a]	Haplotype
Xieqingzao	Anhui	ImV	Hap1
Zhuguang 23	Anhui	ImV	Hap1
78130	Fujian	ImV	Hap1
Aijiaobaimizi	Fujian	ImV	Hap1
Hong 410	Fujian	ImV	Hap1
Jiahezaozhan	Fujian	ImV	Hap1
Longge 113	Fujian	ImV	Hap1
Lucaihao	Fujian	ImV	Hap1
Mancang 515	Fujian	ImV	Hap1
Minghui 86	Fujian	ImV	Hap1
Aijiaonante	Guangdong	ImV	Hap1
Chaoyangzao 18	Guangdong	ImV	Hap1
Fengaizhan 1	Guangdong	ImV	Hap1
Gengxian 89	Guangdong	ImV	Hap1
Guangchang 13	Guangdong	ImV	Hap1
Guangjie 9	Guangdong	ImV	Hap1
Guangluai 4	Guangdong	ImV	Hap1
Guangnongai 1	Guangdong	ImV	Hap1
Guangqiuai	Guangdong	ImV	Hap1
Guichao 2	Guangdong	ImV	Hap1
Hongmeizao	Guangdong	ImV	Hap1
Kejiexuan 17	Guangdong	ImV	Hap1
Qilisimiao	Guangdong	ImV	Hap1
Qinghuaai 6	Guangdong	ImV	Hap1
Qishanzhan	Guangdong	ImV	Hap1
Qiuguiai 11	Guangdong	ImV	Hap1
Sanerai	Guangdong	ImV	Hap1
Shuanggui 1	Guangdong	ImV	Hap1
Shuangzhuzhan	Guangdong	ImV	Hap1
Tangbuai	Guangdong	ImV	Hap1
Teqing	Guangdong	ImV	Hap1
Texianzhan 13	Guangdong	ImV	Hap1
Xianxiaozhan	Guangdong	ImV	Hap1
Yuexiangzhan	Guangdong	ImV	Hap1
Zhaiyeqing 8	Guangdong	ImV	Hap1
Aizizhan	Guangxi	ImV	Hap1
Bo B	Guangxi	ImV	Hap1
Tuanjie 1	Guangxi	ImV	Hap1
Zaogui 1	Guangxi	ImV	Hap1
Yuxian 5	Henan	ImV	Hap1
Ezao 18	Hubei	ImV	Hap1
Huaai 15	Hubei	ImV	Hap1
Ce 64	Hunan	ImV	Hap1
Ce 64-7	Hunan	ImV	Hap1

Table S5 (continued)

Name	Origin	Type[a]	Haplotype
Dongtingwanxian	Hunan	ImV	Hap1
II-32B	Hunan	ImV	Hap1
Jin 23 B	Hunan	ImV	Hap1
V20B	Hunan	ImV	Hap1
Xiangaizao 10	Hunan	ImV	Hap1
Xiangaizao 7	Hunan	ImV	Hap1
Xiangwanxian 13	Hunan	ImV	Hap1
Xiangwanxian 17	Hunan	ImV	Hap1
Xiangwanxian 9	Hunan	ImV	Hap1
Xiangzaoxian 1	Hunan	ImV	Hap1
Xiangzaoxian 14	Hunan	ImV	Hap1
Xiangzaoxian 17	Hunan	ImV	Hap1
Xiangzaoxian 19	Hunan	ImV	Hap1
Xiangzaoxian 24	Hunan	ImV	Hap1
Xiangzaoxian 3	Hunan	ImV	Hap1
Xiangzaoxian 31	Hunan	ImV	Hap1
Xiangzaoxian 6	Hunan	ImV	Hap1
Yuchi 231-8	Hunan	ImV	Hap1
Nanjing 11	Jiangsu	ImV	Hap1
Nanjing 16	Jiangsu	ImV	Hap1
Qinglian 16	Jiangsu	ImV	Hap1
Yangdao 6	Jiangsu	ImV	Hap1
Yangfuxian 5	Jiangsu	ImV	Hap1
Yangfuxian 6	Jiangsu	ImV	Hap1
5450	Jiangxi	ImV	Hap1
754	Jiangxi	ImV	Hap1
Bayiwan	Jiangxi	ImV	Hap1
Ganwanxian 14	Jiangxi	ImV	Hap1
Ganwanxian 19	Jiangxi	ImV	Hap1
Ganzaoxian 26	Jiangxi	ImV	Hap1
Ganzaoxian 37	Jiangxi	ImV	Hap1
Liantangzao 4	Jiangxi	ImV	Hap1
M112	Jiangxi	ImV	Hap1
Nantehao	Jiangxi	ImV	Hap1
Xiujiangzao 9	Jiangxi	ImV	Hap1
80-133	Sichuan	ImV	Hap1
Aituogu 151	Sichuan	ImV	Hap1
Duoxi 1	Sichuan	ImV	Hap1
Fuhui 838	Sichuan	ImV	Hap1
Huhongzao 1	Sichuan	ImV	Hap1
Lunanzao 1	Sichuan	ImV	Hap1
Lushuang 1011	Sichuan	ImV	Hap1
Ainanzao 1	Zhejiang	ImV	Hap1

Table S5 (continued)

Name	Origin	Type[a]	Haplotype
Chaoyang 1	Zhejiang	ImV	Hap1
Erjiufeng	Zhejiang	ImV	Hap1
Erjiuqing	Zhejiang	ImV	Hap1
Huazhan	Zhejiang	ImV	Hap1
Jiayu 293	Zhejiang	ImV	Hap1
Jiayu 935	Zhejiang	ImV	Hap1
Jiayu 948	Zhejiang	ImV	Hap1
Qingganhuang	Zhejiang	ImV	Hap1
Shuangke 1	Zhejiang	ImV	Hap1
Simei 2	Zhejiang	ImV	Hap1
Wenxuanqing	Zhejiang	ImV	Hap1
Xianfeng 1	Zhejiang	ImV	Hap1
Yuanfengzao	Zhejiang	ImV	Hap1
Zhe 76-1	Zhejiang	ImV	Hap1
Zhe 852	Zhejiang	ImV	Hap1
Zhe 9248	Zhejiang	ImV	Hap1
Zhefu 802	Zhejiang	ImV	Hap1
Zhenshan 97	Zhejiang	ImV	Hap1
Zhong 86-44	Zhejiang	ImV	Hap1
Zhong 9B	Zhejiang	ImV	Hap1
Zhongganzao	Zhejiang	ImV	Hap1
Zhongjiazao 17	Zhejiang	ImV	Hap1
Zhongyouzao 3	Zhejiang	ImV	Hap1
Zhongyouzao 81	Zhejiang	ImV	Hap1
Zhongzao 33	Zhejiang	ImV	Hap1
Zhongzao 39	Zhejiang	ImV	Hap1
Zhou 903	Zhejiang	ImV	Hap1
Zhulianai	Zhejiang	ImV	Hap1
Baijiaoe	Anhui	Lr	Hap1
Guangyexian	Anhui	Lr	Hap1
Maotudimeng	Anhui	Lr	Hap1
Muqiuchui	Anhui	Lr	Hap1
Nibuzhan	Anhui	Lr	Hap1
Sanlicun	Anhui	Lr	Hap1
Wujiezaodao	Anhui	Lr	Hap1
Yiluxiang	Anhui	Lr	Hap1
Zhuchaxian	Anhui	Lr	Hap1
Maoxiangzhan	Chongqing	Lr	Hap1
Xiaomaweizhan	Chongqing	Lr	Hap1
Bolizhan	Fujian	Lr	Hap1
Fanganzao	Fujian	Lr	Hap1
Shimaren	Fujian	Lr	Hap1
Yazhan	Fujian	Lr	Hap1
Yuhu	Fujian	Lr	Hap1

Table S5 (continued)

Name	Origin	Type[a]	Haplotype
Dahuanggu	Guangdong	Lr	Hap1
Menjiaheisi	Guangdong	Lr	Hap1
Potouzhong	Guangdong	Lr	Hap1
Puningmalongya	Guangdong	Lr	Hap1
Shanlanzhan	Guangdong	Lr	Hap1
Xianshuizhan	Guangdong	Lr	Hap1
Hantaizhan	Guangxi	Lr	Hap1
Hejian	Guangxi	Lr	Hap1
Shangu	Guangxi	Lr	Hap1
Xinzhenggu	Guangxi	Lr	Hap1
Dishuigu	Guizhou	Lr	Hap1
Lengshuizhan	Guizhou	Lr	Hap1
Mengguandamagu	Guizhou	Lr	Hap1
Qinggangu	Guizhou	Lr	Hap1
Wanminuo	Guizhou	Lr	Hap1
Wumingaixian	Guizhou	Lr	Hap1
Baijiaoheijie	Hainan	Lr	Hap1
Daziben	Henan	Lr	Hap1
Xianzidao	Henan	Lr	Hap1
Yintiaozhan	Henan	Lr	Hap1
Bawangbian	Hubei	Lr	Hap1
Duligan	Hubei	Lr	Hap1
Enshidishuidao	Hubei	Lr	Hap1
Fangxianwubaili	Hubei	Lr	Hap1
Gaoganhe	Hubei	Lr	Hap1
Hongmidongzhan	Hubei	Lr	Hap1
Kuxindao	Hubei	Lr	Hap1
Machengjiangxizao	Hubei	Lr	Hap1
Shuibawang	Hubei	Lr	Hap1
Tedali	Hubei	Lr	Hap1
Xiyedongzhan	Hubei	Lr	Hap1
Ziguilengshuigu	Hubei	Lr	Hap1
Changuzao	Hunan	Lr	Hap1
Chihe	Hunan	Lr	Hap1
Daozhouzao	Hunan	Lr	Hap1
Langanzhan	Hunan	Lr	Hap1
Tiejiaozhan	Hunan	Lr	Hap1
Yapozhan	Hunan	Lr	Hap1
Youzhan	Hunan	Lr	Hap1
Dingyuangu	Jiangsu	Lr	Hap1
Yinghongke	Jiangsu	Lr	Hap1
Bayuebai	Jiangxi	Lr	Hap1
Yingguzhan	Jiangxi	Lr	Hap1
Dalangan	Sichuan	Lr	Hap1

Table S5 (continued)

Name	Origin	Type[a]	Haplotype
Qishizao	Sichuan	Lr	Hap1
Tieganlu	Sichuan	Lr	Hap1
Zhaodaowen	Sichuan	Lr	Hap1
Taizhonghongxuguzi	Taiwan	Lr	Hap1
Babaomi	Yunnan	Lr	Hap1
Baiganlufeng	Yunnan	Lr	Hap1
Bairizao	Yunnan	Lr	Hap1
Haoannongmiemen	Yunnan	Lr	Hap1
Haojingke	Yunnan	Lr	Hap1
Huangbansuo	Yunnan	Lr	Hap1
Lengshuibaigu	Yunnan	Lr	Hap1
Lengshuizao	Yunnan	Lr	Hap1
Niumaoxiangzao	Yunnan	Lr	Hap1
Xigu	Yunnan	Lr	Hap1
Zigangu	Yunnan	Lr	Hap1
Niantianshi	Zhejiang	Lr	Hap1
Dayexiaoxianggu	Shanxi	Lr	Hap1
Zaomiaosi	Shanxi	Lr	Hap1
MA HNAN THU KA	Burma	Ex	Hap1
X72-7-1	Burma	Ex	Hap1
NEANG CHHOUK	Cambodia	Ex	Hap1
NEANG SAN	Cambodia	Ex	Hap1
AMISTAD 82-8	Cuba	Ex	Hap1
C.CORTO 1	Cuba	Ex	Hap1
Gu 2725	Cuba	Ex	Hap1
SELECCION VG-5	Cuba	Ex	Hap1
GIZA 181	Egypt	Ex	Hap1
CSR 11	India	Ex	Hap1
HPA 74	India	Ex	Hap1
JC 195	India	Ex	Hap1
JW 42	India	Ex	Hap1
K 428-25	India	Ex	Hap1
KALING A2	India	Ex	Hap1
NDR 4012	India	Ex	Hap1
RP 1017-76-1-3-2	India	Ex	Hap1
VL DHAN 16	India	Ex	Hap1
B 6397F-MR-7-5M-1-1	Indonesia	Ex	Hap1
BAHBUTONG	Indonesia	Ex	Hap1
BATANG PANE	Indonesia	Ex	Hap1
CIMANUK	Indonesia	Ex	Hap1
CIPUNEGARA	Indonesia	Ex	Hap1
habataki	Japan	Ex	Hap1
Sanjiang	Japan	Ex	Hap1
ITA 304	Nigeria	Ex	Hap1

Table S5 (continued)

Name	Origin	Type[a]	Haplotype
Shuiyuan 299	North Korea	Ex	Hap1
BASMATI 685	Pakistan	Ex	Hap1
GANJAY(ACC76349)	Pakistan	Ex	Hap1
PANAMA 1537	Panama	Ex	Hap1
75-1-120	Philippines	Ex	Hap1
97A-M54	Philippines	Ex	Hap1
PR 23631-98	Philippines	Ex	Hap1
SINABA	Philippines	Ex	Hap1
SINANDOMENG	Philippines	Ex	Hap1
BATHKIRIEL	Sri Lanka	Ex	Hap1
BG 170	Sri Lanka	Ex	Hap1
MALKORA(Acc.11716)	Sri Lanka	Ex	Hap1
MURUNGAWEE	Sri Lanka	Ex	Hap1
SINNA SIVAPPU	Sri Lanka	Ex	Hap1
NIAW SANPAH TAWNG	Thailand	Ex	Hap1
Xianluosichi	Thailand	Ex	Hap1
CHAH NONG NGHE AN	Vietnam	Ex	Hap1
LAI TRANG	Vietnam	Ex	Hap1
QUCM	Vietnam	Ex	Hap1
Yangdao 2	Sri Lanka	Ex+	Hap1
IR36	Philippines	Ex+	Hap1
IR64	Philippines	Ex+	Hap1
Milyang 46	South Korea	Ex+	Hap1
Baokanglengshuihong	Hubei	Lr	Hap2
Baxiancungu	Hubei	Lr	Hap2
Bingshuibai	Hubei	Lr	Hap2
Erlicun	Hubei	Lr	Hap2
Lengshuihong	Hubei	Lr	Hap2
RANGPUR (KANGPUR)	Bangladesh	Ex	Hap2
IB 42	Burundi	Ex	Hap2
IB 28	Burundi	Ex	Hap2
RT 1031-69	Congo	Ex	Hap2
JW 60	India	Ex	Hap2
Boyo	Indonesia	Ex	Hap2
PADI SEGUTUK	Indonesia	Ex	Hap2
Milyang 70	North Korea	Ex	Hap2
Dawn CI9534	USA	Ex	Hap2
Nova 66 CI9481	USA	Ex	Hap2
DOMSIAH 138	Vietnam	Ex	Hap2
T 23	India	Ex	Hap3
Basmati 370	Pakistan	Ex	Hap3
JIJAI NIKI(ACC76358)	Pakistan	Ex	Hap3
RATRIA(ACC28500)	Pakistan	Ex	Hap3
BINICOL	Philippines	Ex	Hap3

Table S5 (continued)

Name	Origin	Type[a]	Haplotype
CHAKULA	Bangladesh	Ex	Hap4
Dular	India	Ex	Hap4
KATAHATA HAMBA	Sri Lanka	Ex	Hap4
Yuxian 3	Henan	ImV	Hap5
Ezao 6	Hubei	ImV	Hap5
Xiangwanxian 1	Hunan	ImV	Hap5
Ganwanxian 30	Jiangxi	ImV	Hap5
Shuhui 527	Sichuan	ImV	Hap5
Taizhongzailai 1	Taiwan	ImV	Hap5
Wubaili	Hubei	Lr	Hap5
Shuangkanghong	Hunan	Lr	Hap5
Jiugongji	Jiangxi	Lr	Hap5
AKHNI SAIL	Bangladesh	Ex	Hap5
BERI	Bangladesh	Ex	Hap5
CSR 12	India	Ex	Hap5
CSR 13	India	Ex	Hap5
CSR 5	India	Ex	Hap5
CSR 9	India	Ex	Hap5
NDR 89	India	Ex	Hap5
OR 79-21	India	Ex	Hap5
PR32-PD47-PD4	India	Ex	Hap5
TM 10265	India	Ex	Hap5
SHINKWANG	Korea	Ex	Hap5
JHALI	Nepal	Ex	Hap5
368	Pakistan	Ex	Hap5
KHARA GANJA(ACC76363）	Pakistan	Ex	Hap5
A1452	Philippines	Ex	Hap5
Pratao Precoce	Philippines	Ex	Hap5
BG 915	Sri Lanka	Ex	Hap5
BG 1165-1	Sri Lanka	Ex	Hap5
BAU HUONG DOONG	Vietnam	Ex	Hap5
CHIEM BAC	Vietnam	Ex	Hap5
RE BAU	Vietnam	Ex	Hap5
RE CHANH	Vietnam	Ex	Hap5
IR8	Philippines	Ex+	Hap5
IR24	Philippines	Ex+	Hap5

[a] ImV, Improved variety in China; Lr, Landrace in China; Ex, Exotic germplasm; Ex+, Exotic germplasm widely used in China.

Table S6. Phenotypic difference between Hap1 and other four haplotypes.

Location	Trait[a]	Phenotype (mean ± SD)[b]				
		Hap1 (242)	Hap5 (33)	Hap2 (16)	Hap3 (5)	Hap4 (3)
Lingshui	HD	93.0 ± 13.2	106.3 ± 14.3 ***	101.8 ± 14.3 **	91.5 ± 3.8	86.3 ± 3.9
	TGW	27.20 ± 4.04	26.66 ± 3.89	28.62 ± 7.56	21.65 ± 1.84 **	24.95 ± 0.21
	GL	8.465 ± 0.858	8.719 ± 0.781	8.970 ± 1.605 *	9.275 ± 0.763 *	8.418 ± 0.372
	GW	3.008 ± 0.306	2.867 ± 0.300 **	3.039 ± 0.311	2.447 ± 0.100 ***	2.980 ± 0.175
Hangzhou	HD	82.3 ± 19.7	85.2 ± 11.4	78.8 ± 8.6	104.5 ± 9.5 **	69.5 ± 2.5
	TGW	25.15 ± 3.67	24.40 ± 3.50	25.50 ± 7.36	18.06 ± 0.41 ***	24.11 ± 1.63
	GL	8.458 ± 0.835	8.604 ± 0.827	8.799 ± 1.532	8.843 ± 0.374	8.546 ± 0.427
	GW	2.774 ± 0.301	2.634 ± 0.298 **	2.904 ± 0.359	2.237 ± 0.092 ***	2.725 ± 0.217

[a] HD, heading date (d); TGW, 1,000-grain weight (g); GL, grain length (mm); GW, grain width (mm).

[b] Significant difference between Hap1 and other four haplotypes was detected by using Student's *t*-test. * $p < 0.05$, ** $p < 0.01$, *** $p < 0.001$. Numbers in the parenthesis after each haplotype indicate the number of varieties.

Table S7. Primers used in this study.

Name	Primer sequence	Purpose
Te20863	F, 5'- GCCGCCTCTACGAGTT -3'	Mapping
	R, 5'- AAAATCACCCGATTACCACAG -3'	
Te20864	F, 5'- CCAAGTTGATTTCTCTCGCAA -3'	Mapping
	R, 5'- CACCTAACAAATTCGGACCT -3'	
Te20873	F, 5'- GGACGTGCCTATGACGA -3'	Mapping
	R, 5'- GCTGCTACTGCATGCTA -3'	
Te20882	F, 5'- CAAGTGCTCGTATAAACGTGAGAC -3'	Mapping
	R, 5'- CCATGCCACATCGTACTTC -3'	
Cri-56A	F, 5'- TGTGTGCCGGAATGGCCTCCTCAAGA -3'	Vector construction
	R, 5'- AAACTCTTGAGGAGGCCATTCCGGCA -3'	
Cri-56B	F, 5'- CAGTGGTCTCAGGCCTGTATGCTTTATAGCGGTCGATGG -3'	Vector construction
	R, 5'- CAGTGGTCTCAAGATCCATCGACCGCTATAAAGCATACA -3'	
Cri-56C	F, 5'- CAGTGGTCTCAGGCCGAAGGATGCTCTATTGAAGAACTG -3'	Vector construction
	R, 5'- CAGTGGTCTCAAGATCAGTTCTTCAATAGAGCATCCTTC -3'	
OE-56	F, 5'- ATCCTCTAGAGTCGAGGAGGAGTAGCGGGTAGCG -3'	Vector construction
	R, 5'- GAGCCCTGGCATGCCTTTGTACTTACCGGAAATTTTATTC -3'	
KO-56A	F, 5'- AAGCGGATTGAGAACCCGAC -3'	Mutation detection
	R, 5'- AGAACTTGCCGAGAACGAGA -3'	
KO-56B	F, 5'- TTTTAGTTTGCTCCTTGTGTCT -3'	Mutation detection
	R, 5'- ACGCAAGAACTCGTCAACA -3'	
KO-56C	F, 5'- CATGATAATCCTGAGAGCCAA -3'	Mutation detection
	R, 5'- TGGCCATTAGGAGTACTGAA -3'	
Seq56-1	F, 5'- CCACCTCCCATCCGCTACTGCT -3'	Sequencing
	R, 5'- TGCCAAATCCGAACAGAGAACCCAT -3'	
Seq56-2	F, 5'- TAGCCACCACTGTTTGTAGCATC -3'	Sequencing
	R, 5'- AGAGCTATATTAACAAACACGCCAT -3'	
Seq56-3	F, 5'- ATTAACTCCATTTTAGCCATGCACT-3'	Sequencing
	R, 5'- TTTGTTCGAAGGCCCAGATTAACCC-3'	
Hyg	F, 5'- GTTTATCGGCACTTTGCATCG -3'	Transgene detection
	R, 5'- GGAGCATATACGCCCGGAGT -3'	
Neo	F, 5'- ACAACTTAATAACACATTGCGGACGTT -3'	Transgene detection
	R, 5'- AATCCCACTATCCTTCGCAAGACCT -3'	
Exon-56	F, 5'- CGCTATAAAGCATACACAAAGGATCA -3'	Transgene detection
	R, 5'- TGGCCAAACCTAAAGTATCATCT -3'	
Actin	F, 5'- TCCATCTTGGCATCTCTCAG -3'	qRT-PCR
	R, 5'- GTACCCGCATCAGGCATCTG -3'	
qRT-56	F, 5'- AGCCTCCACAACGATAAGACTAAAGAAGG -3'	qRT-PCR
	R, 5'- GCAGCCTCAAGGTTGCGATG -3'	
qRFT1	F, 5'- TGACCTAGATTCAAAGTCTAATCCTT -3'	qRT-PCR
	R, 5'- TGCCGGCCATGTCAAATTAATAAC -3'	
qHd3a	F, 5'- GCTCACTATCATCATCCAGCATG -3'	qRT-PCR
	R, 5'- CCTTGCTCAGCTATTTAATTGCATAA -3'	
